# Dental Disease Outcomes Following a 2-Year Oral Health Promotion Program for Australian Aboriginal Children and Their Families: A 2-Arm Parallel, Single-blind, Randomised Controlled Trial

**DOI:** 10.1016/j.eclinm.2018.05.001

**Published:** 2018-07-23

**Authors:** Lisa Jamieson, Lisa Smithers, Joanne Hedges, Eleanor Parker, Helen Mills, Kostas Kapellas, Herenia P. Lawrence, John R. Broughton, Xiangqun Ju

**Affiliations:** a Adelaide Dental School, University of Adelaide, Australia; b School of Public Health, University of Adelaide, Australia; c School of Dentistry, University of Toronto, Canada; d School of Dentistry, University of Otago, New Zealand

**Keywords:** Early childhood caries, Aboriginal, Randomised controlled trial, Oral health promotion

## Abstract

**Background:**

Dental disease has far-reaching impacts on child health and wellbeing. We worked with Aboriginal Australian communities to develop a multifaceted oral health promotion initiative to reduce children's experience of dental disease at age 2 years.

**Methods:**

This was a single-blind, parallel-arm, randomised controlled trial. Participants were recruited from health service providers across South Australia. Women pregnant with an Aboriginal child were eligible. The intervention comprised: (1) provision of dental care to mothers during pregnancy; (2) application of fluoride varnish to teeth of children at ages 6, 12 and 18 months; (3) motivational interviewing delivered in conjunction with; (4) anticipatory guidance. The primary outcome was untreated dental decay as assessed by the number of teeth with cavitated and non-cavitated carious lesions (mean dt) at child age 24 months. Analyses followed intention-to-treat principles. The RCT was registered with the Australian and New Zealand Clinical Trial Registry, ACTRN12611000111976.

**Findings:**

Women (n = 448) were recruited from February 2011 to May 2012, resulting in 223 children in the treatment group and 225 in the control. Mean dt at age two years was 0.62 (95% CI 0.59 to 0.65) for the intervention group and 0.89 (95% CI 0.85 to 0.92) for the control group (mean difference − 0.27 (95% CI − 0.31, − 0.22)).

**Interpretation:**

A culturally-appropriate intervention at four time-points from pregnancy through to 18-months resulted in improvements in the oral health of Aboriginal children. Further consultation with Aboriginal communities is essential for understanding how to best sustain these oral health improvements for young Aboriginal children.

**Research in Context**

**Evidence before this study**

We searched Pubmed, EMBASE, Dentistry and Oral Sciences Source (DOSS via EBSCO host) and the Cochrane library from inception until December 2017 using the following search strategy; (aborigin* OR indigenous) AND (infant OR child OR toddler OR parent* OR family) AND (motivational interview* OR anticipatory guidance OR fluoride varnish OR dental care pregnancy). We also searched the Australian Indigenous HealthInfoNet website (www.healthinfonet.ecu.edu.au) from inception in 2010 until November 2017. The HealthInfoNet website is dedicated to disseminating evidence on Australian Aboriginal and Torres Strait Islander health. The sections of the website searched were the Publications (Oral health, Childhood growth, and Infants and young children subsections) and the Programs and Projects (Infants and Oral health subsections). After screening all titles and summaries, only studies involving interventions among families with children < 5 years and had evaluated dental disease outcomes were considered further.

Among non-Aboriginal parents, a systematic review of studies involving motivational interviewing suggests that there are benefits to oral health outcomes of children (*k* = 16 studies), however the evidence base is small for interventions involving children < 2 years of age (only *k* = 3 studies) [43]. One Cochrane Collaboration systematic review examining community-based interventions for oral health concluded that there are too few RCTs or a lack of robust evidence that home visiting, motivational interviewing, or strategies involving dental education or tooth brushing with fluoride toothpaste are beneficial for promoting child oral health [44]. To date, and to the best of our knowledge, there have been two randomised controlled trials (RCTs) that have assessed the effectiveness of the application of fluoride varnish on reducing dental disease among Indigenous children; one in Canada [14] and the other in Australia [15]. There has been one RCT that has demonstrated the effectiveness of anticipatory guidance in reducing prevalence of carious lesions among young children in the general Australian population [17]. There have been no RCTs that have assessed the role of motivational interviewing in preventing dental disease among young Aboriginal children. In a 2013 Cochrane Collaboration systematic review of fluoride varnish, the pooled estimate of prevented fraction in the teeth of young children was 37% (95% CI 24% to 51%) [36].

**Added value of this study**

The current study provides the only evidence of a large, multi-faceted oral health promotion intervention among Aboriginal children in the first two years of life that has been; 1) developed with Aboriginal communities, 2) tested in a randomised design, and 3) **involved** comprehensive dental disease outcomes. Our culturally-tailored intervention demonstrated tangible improvements to children’s experience of dental disease, particularly when children residing in non-metropolitan settings were considered in isolation. Our data add valuable evidence to the understanding of motivational interviewing, anticipatory guidance, application of fluoride varnish and provision of dental care to mothers during pregnancy on Aboriginal child oral health; findings that may be translated to Indigenous groups in other countries and settings.

**Implications of all the available evidence**

Implementation of a multi-faceted oral health promotion intervention may only reduce Aboriginal children’s experience of dental diseases if there are concomitant changes in the social determinants that shape behaviours leading to dental disease progression in the first place. A sugar-sweetened beverage tax may be beneficial in reducing consumption of products with notable associations with dental disease (among both adults and children). Engaging with Aboriginal community-controlled health organisations and promoting a specific ‘specialty’ in oral health in current Aboriginal Health Worker training packages may increase education opportunities to promote positive oral health behaviours that, in turn, may facilitate the types of improvements in dental health-related behaviours that are needed to substantially affect Aboriginal oral health at a population level.

Alt-text: Unlabelled Box

## 1 Introduction

Oral health is a fundamental human right. Poor oral health, particularly when related to untreated carious lesions, contributes substantially to general morbidity, to household economic stress and to poor quality of life [1]. In many developed countries, untreated carious lesions are the leading cause of preventable child hospital admissions, usually for treatment under a general anaesthetic [2], [3]. Dental caries in childhood is the strongest predictor of dental caries in adulthood [4]. Throughout the world, Indigenous children experience high levels of carious lesions, generally much higher than that experienced by their non-Indigenous peers. In the United States, Braun and colleagues reported that 88% of Navajo children (mean age 3.7 years) involved in their study had carious lesions at baseline, which increased to almost 100% at the 3-year follow-up [5]. In Canada's First Nations Oral Health Survey (2009–10), 86% of children aged 3 to 5 years had dental caries experience [6], while in the New Zealand National Oral Health Survey (2009), the prevalence of untreated carious lesions in the primary dentition of Maori children aged 2 to 11 years was 27%, twice that reported for non-Maori children after adjusting for age and sex [7]. Dental disease experience among the Aboriginal and Torres Strait Islander child population in Australia is also high; in Australia's most recent National Child Oral Health Survey (2012–14), 44% of Aboriginal children aged 5 to 10 years had untreated carious lesions, while it was 26% among non-Indigenous children [8].

The literature suggests a number of ways in which early childhood caries (defined as the presence of one or more decayed, missing (due to caries) or filled tooth surfaces in any primary tooth in a child 71 months of age or younger) [9] can be successfully prevented. After consultation with the South Australian Aboriginal community, we have selected four: (1) dental care provided to the mother during pregnancy; (2) fluoride varnish application to the teeth of children; (3) anticipatory guidance; and (4) motivational interviewing. Provision of comprehensive dental care to mothers during pregnancy reduces maternal levels of *Streptococcus mutans* [10], [11], [12]. *Streptococcus mutans* is a commensal bacterium in oral biofilm that thrives when frequently exposed to fermentable carbohydrate, further exacerbated when oral hygiene habits are poor. The application of topical fluoride varnish to the teeth of infants and pre-school children has been shown to be efficacious in the prevention of early childhood caries, with Weintraub and colleagues reporting little difficulty with adherence and no adverse events [13]. The application of fluoride varnish has also been reported to prevent dental caries among First Nation children in Canada [14] and among Aboriginal children in Australia [15]. Anticipatory guidance is a pro-active, developmentally-based counselling technique that focuses on the needs of a child at a particular stage of life [16]. Plutzer and Spencer demonstrated how an anticipatory guidance intervention was associated with fewer carious lesions among children in an RCT [17], with the effects being sustained until age 6–7 years [18]. Motivational interviewing, on the other hand, focuses on strategies to move carers from inaction to action, with many possible paths provided to a solution [19]. In a recent review, Finlayson reported that motivational interviewing may be the most effective behavioural intervention for reducing dental caries in children [20]. To date, there have been no reports of an initiative that has adopted all intervention strategies simultaneously. When provided in a culturally-appropriate way, a strategy that employs all four interventions could provide a foundation for preventive dental care and oral health education, and thus enhance the opportunity for an Indigenous childhood free from oral disease.

Given that no child is born with carious lesions, a logical step is to implement an early childhood caries intervention among woman pregnant with an Aboriginal child, including elements of the four interventions outlined above, beginning during pregnancy and taking place throughout early childhood. The study aim was to determine the efficacy of a four-pronged early childhood caries intervention among Aboriginal Australian children. We hypothesised that a context-specific, multi-faceted early childhood caries intervention would prevent development of carious lesions among this vulnerable population.

## 2 Methods

### 2.1 Study Design and Participants

We conducted a single-blind randomised controlled trial that was developed in partnership with local Aboriginal communities and endorsed by the study's Aboriginal Reference Group. Consultation with the South Australian Aboriginal community was done through 3 focus group discussions (one metropolitan, two regional) and via individual consultation with members of the study's Aboriginal Reference Group. Participants were 448 women pregnant with an Aboriginal child and who were residing in South Australia in the recruitment period of February 2011 to May 2012. The sample represented two-thirds of those who were eligible during the recruitment period, and was representative by age, socio-economic position and tobacco smoking status [21]. The study received approval from the University of Adelaide Human Research Ethics Committee (H-057-2010), the Aboriginal Health Council of South Australia (04-09-362), the Government of South Australia and the Human Research Ethics Committees of the three participating South Australian birthing hospitals. The trial is registered with the Australian New Zealand Clinical Trial Registry (ACTRN12611000111976) and the protocol has been published [22].

### 2.2 Randomisation and Masking

The randomisation schedule was prepared by a statistician not otherwise involved in the trial, using a computerised random number generator and random block sizes of 4, 6 and 8. The randomisation schedule was stratified by metropolitan and non-metropolitan recruitment area. Participants were randomly assigned to either the intervention or control group (1:1 ratio). Allocation to a group occurred through a central randomisation service via a computer algorithm, which protected the randomisation schedule. Because of the nature of the intervention, neither participants nor research officers who implemented the intervention were blind to the treatment allocation. However, to achieve single blinding, a separate set of research staff collected 2-year outcome data.

### 2.3 Procedures

There were four components to the early childhood caries intervention, underpinned by evidence in the literature demonstrating each method's efficacy in reducing early childhood caries. The four modalities were selected based on their acceptance in the Aboriginal community and the ability to deliver outcomes in the time-frame of the funded RCT.

1. Provision of dental care to mothers during pregnancy: Mothers randomly allocated to the intervention arm who owned a Government means-tested health care card were eligible for public dental care through South Australia's Dental Service (SADS). Appointments and transport were organised by study staff, with assistance from the SADS Aboriginal Liaison Program. Participants not eligible for publicly-funded dental care had dental treatment organised through one of six private dental providers who were partners in the project. No costs were incurred by study participants for either the public or privately provided dental care. Dental care was comprehensive in its remit, and included check-ups, X-rays, dental scaling and cleanings, restorations and extractions (including wisdom teeth). Orthodontics, endodontics and cosmetic dentistry were not provided. If endodontic care was required, participants needed to pay out-of-pocket for this treatment.
2. Application of fluoride varnish to the teeth of the children at age 6, 12 and 18 months: The fluoride varnish protocol was adapted from that used by Slade and colleagues [15] and was implemented by study staff who had been trained in its use. Briefly, the knee-to-knee position was adopted, with the child's head on the lap of study staff. Children's teeth were then cleaned and dried with gauze, and fluoride varnish applied beginning with the posteriors (if present) and moving anteriorly. Carers were advised to refrain from giving the child food or drink for half an hour. The guidelines used when fluoride use was discussed for children aged 12 months and younger were based on those developed by the South Australian Dental Service. Fluoride varnish was not applied if a child had no tooth/teeth at age 6 months.
3. Anticipatory Guidance. Anticipatory guidance is the process through which practical information which is both tailored and developmentally-appropriate is delivered to parents in an individualised way (that is, taking into account the parents' specific health concerns, financial barriers and social support structures), so as to better prepare them for important physical, emotional, and psychological milestones of their child as they relate to oral health [23]. In our study, tailored oral health educational packages were compiled with dental-specific information relevant for mothers during pregnancy (focus on dental care provision, pregnancy gingivitis) and when children were aged 6 months (focus on first solid foods, caring for infant teeth when they first erupt), 12 months (focus on tooth brushing and fluoride, avoiding sugar-containing foods and beverages) and 18 months (focus on baby's first dental check-up, eruption of molar teeth).
4. Motivational interviewing (MI) was implemented in combination with the anticipatory guidance; that is, with mothers during pregnancy and when children were aged 6, 12 and 18 months. Study staff attended a basic two-day MI training course, followed by an intense one-day follow-up course. Monthly one-day follow-up training was continued for six months, followed by bi-monthly one-day coaching, and occasional ad-hoc telephone coaching, for another year. The sessions were tailored to suit the needs of individual participants, with each session conducted on a one-to-one basis in participants' homes or other venues where participants felt comfortable (for example, community halls or local Aboriginal health services). The duration of each session ranged from 30 to 90 min. Plain English and pictorial prompts were utilised, as recommended [24]. Fidelity was assessed by a member of the Motivational Interviewing Network of Trainers, and found to be acceptable [25].

### 2.4 Outcomes

The primary outcome was initially slated as being child dental caries experience (the mean number of decayed, missing or filled teeth; dmft). However, no children had missing or filled teeth at age two years, so the primary outcome was revised to mean number of decayed teeth (dt). This comprised teeth with cavitated or non-cavitated carious lesions. The information was collected during standardised examinations conducted at 24-month follow-up by three calibrated dental professionals (intra-class correlations for mean dt between each examiner and the gold standard examiner ranged from 0.80 to 0.88). These staff followed a standardised protocol to record dental disease experience. Procedures appropriate for young children were used, for example, children were examined in the ‘knee-to-knee’ position on their carer's lap. Before the examination, teeth were dried with cotton pads. Standard infection control procedures were followed and a fibre-optic light used as a light source. Diagnosis was based on visual criteria only. Carious lesions were computed at the threshold of both pre-cavitation and cavitation. Pre-cavitation is considered to be an area of demineralization without loss of surface continuity, while cavitation is defined as a visible break in the enamel surface caused by dental caries. Any child diagnosed with carious lesions was referred for dental care through the South Australian Dental Service (provided free of charge).

### 2.5 Baseline Descriptive Variables

Baseline descriptive variables included socio-demographic characteristics (maternal age, education, income, means-tested health care card status and residential location), health status and dental behaviours (usual reason for visiting a dentist, maternal tooth brushing behaviour, self-rated oral health and self-rated general health).

Maternal age was categorised into ‘14–24 years’ or ‘25 + years’, while education was dichotomized into ‘high school or less’ or ‘trade/technical or University’. Income was dichotomized into ‘job’ or ‘Centrelink’ (welfare) and ownership of a means-tested Government Health Care Card (yes/no). Centrelink is the Australian agency which provides welfare payments to those who are unemployed. Residential location was dichotomized into ‘metropolitan’ (Adelaide and outer suburbs) and ‘non-metropolitan’ (regional areas).

Self-rated general/oral health status was obtained from asking: ‘How do you think your general/dental health is?’. Responses were categorised into ‘excellent, very good, good’ vs ‘fair, poor’. Dental behaviours included the question ‘Did you brush your teeth yesterday?’ with response options including ‘yes’ or ‘no’; and ‘What is your usual reason for seeing a dentist?’. Response options included ‘problem’ or ‘check-up’.

### 2.6 Sample Size

Based on a recent Aboriginal early childhood caries intervention conducted in Australia's Northern Territory-dwelling Indigenous children [15], it was estimated that a sample size of 280 (140 in each arm of the trial) would be necessary to detect a 25 per cent difference in early childhood caries prevalence between the two groups, at the significance criterion of 0.05 and a power of 0.80. The literature indicates that it is reasonable to expect a difference in effect of this magnitude following a motivational interviewing intervention among Indigenous children [19]. Allowing for an attrition rate of 35% after 36 months, 436 participants would be necessary at base-line; rounded up to 440 for convenience (220 intervention group, 220 control group).

### 2.7 Statistical Analysis

Data analyses were conducted under intention-to-treat principles. The number and percentage of participant characteristics were calculated for both intervention and control groups. Baseline data was used to generate descriptive statistics for the intervention and control groups. General linear regression models were used to compare the efficacy of the intervention on early childhood caries (mean dt) between intervention and control groups, adjusting for baseline maternal socio-demographic, health status and dental behaviour characteristics. The models' least squares means provided adjusted dt per child and associated 95% confidence intervals (CIs) for intervention and control groups. The measure of intervention efficacy was the difference between intervention and control groups in adjusted dt per child. This efficacy estimate represents the average number of teeth, per child, in which dental caries was prevented as a result of the intervention. The prevented fraction is the percentage of cases that can be prevented if a population is exposed to an intervention, compared to an unexposed population. It was calculated by dividing the absolute value of the efficacy estimate by the mean of the related dependent variable in the control group. In our study, this was the number of carious lesions that could be prevented if the population is exposed to the intervention compared to an unexposed population. Treatment effect was additionally measured using number of children needed to treat (NNT) analysis to determine how many children need to be exposed to the intervention to prevent one child from developing new carious lesions (non-cavitated + cavitated lesions). Due to unequivocal evidence from the literature reporting differences in Indigenous child caries rates between residential areas [32], [33], [34], [35], it was decided a priori to conduct subgroup analyses by metropolitan and non-metropolitan location. Missing data was imputed under the assumption that data was missing at random (MAR) using the Fully Conditional Specification (FCS) method with logistic regression for binary variables and linear regression for continuous variables. All missing data were imputed (outcomes and baseline variables) within intervention and control groups separately, except outcomes for deceased infants which were not imputed. We created 50 imputed datasets using 50 iterations, with the results from the imputed datasets combined using Rubin's rules via the ‘Proc mianalyse’ function. The imputed analyses are the primary results. Sensitivity analyses were conducted by using the ‘MNAR adjust statement’ with different scenarios, which included different percentages of MAR assumptions and maximum and minimum value imputations, to ensure validity of the multiple imputation (MI) process. Sensitivity analysis findings were consistent across all models. The SAS statistical software (SAS 9.4, SAS Institute Inc., Cary, NC, USA) was used to impute and analyse data.

### 2.8 Role of the Funding Source

The study funders had no role in study design; in the collection, analysis and interpretation of data; in writing of the report; and in the decision to submit the paper for publication. LMJ, LGS and XJ had full access to all the data in the study. LMJ had final responsibility for the decision to submit for publication.

## 3 Results

**Fig. 1:**
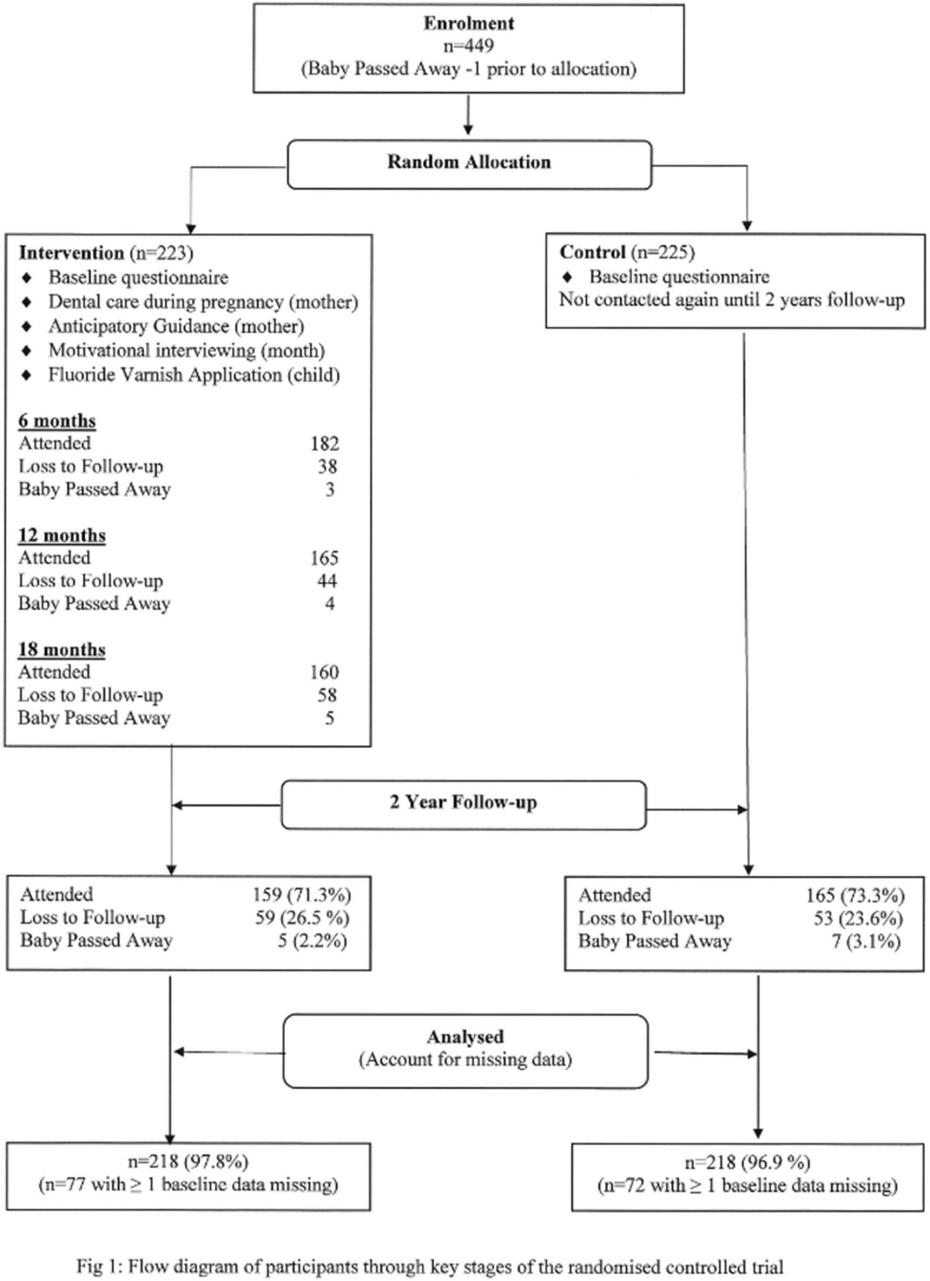
Flow diagram of participants through key stages of the randomised controlled trial.

Four hundred and forty eight women pregnant with an Aboriginal child who completed an early childhood caries-related questionnaire at baseline were randomly allocated to either the intervention (n = 223) or control group (n = 225) (Fig. 1). At the two-year follow-up, 325 mother–child dyads were retained; 159 intervention (71.3%) and 165 control (73.3%). Child mean age was 2.22 years (95% CI: 2.17, 2.27) in the intervention group and 2.23 years (95% CI: 2.19–2.28) in the control group. Between the baseline and two-year follow-up, 12 babies had passed away and 112 participants were lost to follow-up (59 from intervention and 53 from control). For the intention-to-treat analysis (missing data imputed), data were available for 218 in the intervention group and 218 in the control group (data on deceased infants not imputed).

The sample characteristics at baseline are summarised in Table 1. The groups were similar on all measured characteristics except for self-rated oral health, where a higher proportion of mothers in the intervention group rated their oral health as ‘fair or poor’ (60%) than in the control group (50%).

**Table 1:** Baseline maternal socio-demographic, dental behavioural and psychosocial characteristics by intervention group.

	Total	Intervention	Control
	Baseline number (%)		
Total	448	223	225
Maternal age			
14–24	238 (53.1)	130 (58.3)	108 (48.0)
25 +	210 (46.9)	93 (41.7)	117 (52.0)
Education			
High school or less	322 (72.4)	162 (73.3)	160 (71.4)
Trade or University	123 (27.6)	59 (26.7)	64 (28.6)
Income			
Job	62 (14.0)	32 (14.5)	30 (13.5)
Centrelink	381 (86.0)	189 (85.5)	192 (86.5)
HCC status			
Yes	358 (82.2)	175 (81.8)	183 (82.8)
No	77 (17.8)	39 (18.2)	38 (17.3)
Residential location			
Metropolitan	171 (38.7)	79 (35.9)	92 (41.4)
Non-metropolitan	271 (61.3)	141 (64.1)	130 (58.6)
Usual reason visit dentist			
Problem	275 (64.0)	141 (65.0)	134 (62.9)
Check-up	155 (36.1)	76 (35.0)	79 (37.1)
Brush yesterday			
Yes	321 (75.01)	158 (74.2)	163 (75.8)
No	107 (25.01)	55 (25.8)	52 (24.2)
Self-rated oral health			
Ex, very good, good	203 (45.3)	90 (40.4)	113 (50.2)⁎
Fair or poor	245 (54.7)	133 (59.6)	112 (49.8)
Self-rated general health			
Ex, very good, good	402 (89.9)	197 (88.7)	205 (91.1)
Fair or poor	45 (10.1)	25 (11.3)	20 (8.9)

⁎ p < 0.05.

The characteristics of children retained in the study at child age 2 years compared to those lost to follow-up are summarised in the Supplementary table. Compared to children retained in the study, there was a higher proportion of mothers in the lost to follow-up group who had received ‘high school or less’ education, relied on welfare for income, had a Government Health Care Card, resided in metropolitan locations, last visited a dentist because of a problem, had not brushed their teeth the previous day and self-rated both their oral health and general health as ‘fair or poor’. However, the maternal characteristics of follow-up and lost-to-follow-up participants were similar across intervention and control groups.

The mean number of decayed teeth at child age two years was lower in the intervention group (0.62) than the control group (0.89) (Table 2). Estimates from the unadjusted model demonstrated that the intervention group had 0.27, 0.07 and 0.19 lower levels of non-cavitated + cavitated lesions, non-cavitated lesions, and cavitated lesions respectively, compared with the control group. The prevented fraction in the unadjusted model was 30.2, 21.9 and 35.3 for non-cavitated + cavitated lesions, non-cavitated lesions, and cavitated lesions respectively. After adjusting for baseline maternal characteristics, health status and dental behaviour characteristics, the efficacy estimate increased slightly to 0.35, 0.09 and 0.26 for non-cavitated + cavitated lesions, non-cavitated lesions, and cavitated lesions respectively, with a prevented fraction of 50.1, 28.5 and 70.1% respectively. Irrespective of the intervention group, metropolitan-dwelling children had significantly less dental caries than their non-metropolitan dwelling counterparts at child age two years. Among children living in non-metropolitan areas, the mean dt of those in the intervention group was significantly less than those in the control group (0.66 compared with 1.13; a mean difference of 0.44 teeth, with a prevented fraction of 39.9%, which increased to 56.5% after adjusting for baseline maternal characteristics). The proportion of children with any caries (per cent non-cavitated + cavitated lesions > 0) was 4% lower in the intervention group compared with the control group.

**Table 2:** Mean number of decayed teeth at child age 2 years by intervention group and residential location.

	Intervention (95% CI)	Control (95% CI)	Unadjusted		Adjusted^b^	
			Mean difference (95% CI)	Prevented fraction (%)	Mean difference (95% CI)	Prevented fraction (%)
Mean dt						
Non-cavitated + cavitated lesions (dt)^a^	0.62 (0.59, 0.65)	0.89 (0.85, 0.92)	− 0.27 (− 0.31, − 0.22)	30.2	− 0.35 (− 0.40, − 0.31)	50.1
Non-cavitated lesions	0.26 (0.25, 0.28)	0.34 (0.19, 0.40)	− 0.07 (− 0.10, − 0.05)	21.9	− 0.09 (− 0.11, − 0.07)	28.5
Cavitated lesions	0.36 (0.34, 0.38)	0.55 (0.52, 0.58)	− 0.19 (− 0.23, − 0.16)	35.3	− 0.26 (− 0.29, − 0.21)	70.0
Mean dt by residential location						
Metropolitan	0.54 (0.50, 0.58)	0.58 (0.55, 0.62)	− 0.04 (− 0.10, 0.01)	7.2	− 0.11 (− 0.17, − 0.06)	25.8
Non-Metropolitan	0.66 (0.63, 0.70)	1.13 (1.05, 1.16)	− 0.44 (− 0.51, − 0.37)	39.9	− 0.54 (− 0.61, − 0.48)	56.5


	Intervention (95% CI)	Control (95% CI)	Unadjusted		Adjusted^b^	
			Risk difference in proportion (95% CI)	Prevented fraction (%)	Risk difference in proportion (95% CI)	Prevented fraction (%)
Percentage of children with						
Non-cavitated + cavitated > 0	19.7 (18.9, 20.4)	23.6 (22.8, 24.4)	− 3.92 (− 5.00, − 2.84)	16.6	− 3.20 (− 6.75, 0.95)	13.6

a Primary outcome.

b Adjusted for baseline maternal socio-demographic, health status and dental behaviour characteristics.

The prevalence of untreated dental caries and number needed to treat (NNT) at age 2 years is presented in Table 3. For all children, the number needed to treat to prevent one child from developing caries (non-cavitated + cavitated lesions) was 25.0. This ranged from 8.9 (mothers with fair or poor self-rated oral health at baseline) to 99 (children living in metropolitan areas).

**Table 3:** Caries prevalence (% dt > 0) and number of children needed to treat (NTT) at 2 years follow-up.

	Intervention (%)	Control (%)	p-Value	NNT
All children	19.7	23.6	< 0.0001	25.0
Maternal age				
14–24	23.6	26.7	0.0005	32.8
25 +	16.5	21.3	< 0.0001	21.1
Education				
High school or less	21.4	26.4	< 0.0001	21.1
Trade or University	17.6	17.6	0.8392	–
Income				
Job	13.7	8.3	< 0.0001	18.4
Centrelink	21.4	26.5	< 0.0001	19.7
HCC status				
Yes	21.1	25.4	< 0.0001	23.3
No	16.3	14.9	0.2498	73.5
Residential location				
Metropolitan	19.0	20.0	0.6232	99
Non-metropolitan	20.9	26.6	< 0.0001	17.5
Usual reason visit dentist				
Problem	20.4	25.7	< 0.0001	18.8
Check-up	20.0	21.3	0.1539	76.9
Brush yesterday				
Yes	18.6	20.3	0.0040	61.7
No	26.3	34.6	< 0.0001	12.2
Self-rated oral health				
Excellent, very good or good	19.8	15.5	< 0.0001	23.1
Fair or poor	21.2	32.4	< 0.0001	8.9
Self-rated general health				
Excellent, very good or good	21.2	24.1	< 0.0001	34.7
Fair or poor	12.0	21.1	< 0.0001	11.0

## 4 Discussion

Our findings suggest that a highly structured, standardised, carefully implemented and culturally-sensitive multifaceted early childhood caries intervention was effective in reducing carious lesions in this Aboriginal child population in an epidemiological sense, but the translation to dental public health settings (where there is usually not the same resources available or rigour applied) may not yield such results. Depending on the analytic assumptions, the intervention reduced net caries increment by 0.27 to 0.35 teeth per child (0.44 to 0.54 teeth per child residing in non-metropolitan locations). This represented 30.2 to 50.1 per cent fewer teeth per child that developed carious lesions over 2 years (39.9 to 56.5 per cent fewer teeth for children in non-metropolitan areas). The intervention was especially efficacious among non-metropolitan dwelling children, suggesting that, if resources are scarce, this might be one group that benefits from tailored and targeted oral health promotion initiatives. The greater exposure to dental caries-related risk factors for Aboriginal children residing in regional or remote locations in Australia, and concomitant higher rates of dental disease, has been well documented. Such risk factors include greater social deprivation (particularly with housing and general community infrastructure), poorer oral health in the Aboriginal adult population (meaning poor oral health in children is sometimes considered the norm), reduced access to dental services and, in some instances, limited purchasing power to access fluoridated toothpastes and toothbrushes [32], [33], [34], [35].

The prevented fraction estimates in our study were higher than those reported by Slade and colleagues for an RCT that aimed to reduce early childhood caries among Aboriginal children in Australia's Northern Territory through application of fluoride varnish and oral health promotion (PF in unadjusted model = 31%, ranging from 24% to 36% after adjusting for community and child risk factors) [15] and for an RCT among Aboriginal children in Canada that focused on application of fluoride varnish and care-giver counselling (preventive fraction = 18%, ranging from 12% to 51% in adjusted models) [14]. In our study, the net effects of the intervention were higher (especially for the non-metropolitan group) than those reported in a 2013 systematic review of fluoride varnish, where the pooled estimate of prevented fraction in primary teeth was 37% (95% CI 24% to 51%) [36].

Our NNT estimates were in the range of those reported by Lawrence and colleagues in an RCT to reduce early childhood caries among First Nation children in Canada (NNT = 12.2, ranging from 7.4 for children aged 4 to 5 years, to 33.3 for those with dfs of 5 +) [14]. Our NNT estimates were also in the range of those reported for an anticipatory guidance-based RCT to reduce early childhood caries among children in South Australia (NNT = 14, 95% CI 10 to 33) [17].

Oral health-related knowledge (such as that provided through the motivational interviewing and anticipatory guidance components of our intervention) is widely acknowledged as being associated with both adult and child oral health behaviours and outcomes [37], indicating that improvements in knowledge may translate into long-term behaviour change that supports oral health. In their review of health literacy-related interventions and outcomes, Berkman and colleagues [38] reported that important components of effective interventions appeared to be their high intensity, theoretical underpinnings, emphasis on skill building, and delivery of the intervention by appropriate staff. Successful interventions appeared to affect intermediate factors, such as increasing knowledge or self-efficacy, or by changing behaviour. Although not successful in all outcomes, our findings, on the whole, reflect these strengths. However, Kay and Locker [39] criticised the sustained benefit of short-term interventions, so the true benefit of the initiative may only be realised if longer-term ‘refreshers’ for study participants are maintained. It could be that follow-up at later ages will be necessary to determine the true, sustained effect of the intervention. However, ongoing contact with the study participants may have resulted in unintended benefits for the control group, meaning longer-term comparisons may need to involve South Australian children not involved in the study.

The overall prevalence and severity of untreated carious lesions among our Aboriginal child participants at age 2 years was high; around 22% of participants had caries prevalence, while the severity (mean number of decayed teeth) was 0.75. It is difficult to compare these findings with population estimates, because information regarding prevalence of early childhood caries among children younger than 4 years is not routinely collected in child dental health surveys in Australia. However, Wyne [40] reported that the prevalence of dental disease experience was 3% among two to three year olds in South Australia, while Plutzer and Spencer [17] reported a prevalence of 4% in their examination of South Australian children aged 20 months. Our estimates are, however, much lower than those reported among Indigenous children elsewhere in Australia and, indeed, at an international level. For example, in a study of 16-month-old American Indian children, Warren and colleagues [41] reported caries prevalence of 32%, with nearly 3% of all erupted tooth surfaces being affected. In the 2010 Indian Health Service Oral Health Survey of American Indian and Alaskan Native Preschool Children [42], 21% of 1-year-olds had carious lesions and 44% of two year olds. The relatively widespread availability of fluoridated water in South Australia may be one reason why estimates of dental disease experience in our study were substantially lower than those reported for Indigenous children elsewhere.

This study has two main strengths. The first is the use of a randomised controlled trial to elicit efficaciousness of an early childhood caries intervention. The second is the deep and sustained involvement of the Aboriginal community in which the intervention was embedded, which resulted in a very good retention rate. Although slightly lower than the retention rates reported by Lawrence and colleagues in Canada (75%) [14], Slade and colleagues in the Northern Territory of Australia (86%) [15] and Braun and colleagues in the United States (83%) [5], it is important to emphasise that participants in our study were younger and not living in designated Aboriginal communities per se, as was the case with the other studies. This means participants are much less confined by geographic and social barriers, and indeed have higher mobility.

Because of the multi-faceted nature of our intervention, it was not possible to determine whether any single component had greater efficacy in reducing children's early childhood caries than another. Our objective was, rather, to obtain the maximum possible benefit for the children through a program of intervention benefits. Although it certainly appears that children in our study have far fewer carious lesions than their Indigenous counterparts, both elsewhere in Australia and internationally, it is only through comparison with their age-matched South Australian cohorts not involved in the trial that we will be able to truly elicit trial efficacy. We aim to do this in the future. An additional limitation is that our intervention included only four of the many ways early childhood caries can be prevented. We did not, for example, include tooth brushing with fluoride toothpaste, community water fluoridation, dental sealants or silver nitrate/silver diamine fluoride.

In conclusion, our multifaceted study of multiple recommended primary preventions for caries in young children, which was adapted to be suitable for the cultural group involved, found improvement over the control group in a statistical sense.

The following is the supplementary data related to this article.

Supplementary table Baseline sample characteristics by follow-up and loss-to-follow-up children at 2-year examinations. Supplementary table

## Contributors

LMJ conceived the idea, obtained funding for the RCT, designed the intervention with local communities, and trained and supervised the collection of dental and related data, with the support of EP, HL and JB. LGS provided critical input on statistical analysis and manuscript writing. JH implemented the intervention, while KK and HM conducted the clinical dental examinations. XJ conducted the statistical analysis and contributed to writing manuscript drafts. The full article was written by LMJ with input from all co-authors. LMJ and LGS are guarantors for this article. All authors read and approved the final version.

## Declaration of Interests

Colgate Palmolive donated toothpaste, toothbrushes, dental floss and disclosing tablets that were offered as a ‘thank you’ package to all participants and their families. Colgate Palmolive and the funding agency, Australia's National Health & Medical Research Council, were not involved in any study design, data collection, analysis or interpretation.

The authors declare they have no financial and personal relationships with other people or organisations that might inappropriately influence this work.

## References

[1] Kassebaum N.J., Bernabé E., Dahiya M., Bhandari B., Murray C.J., Marcenes W. (2015). Global burden of untreated caries: a systematic review and metaregression. J Dent Res.

[2] Public Health England (2016). Tackling poor oral health in children.

[3] Canadian Institute for Health Information (2013). Treatment of preventable dental cavities in preschoolers.

[4] Isaksson H., Alm A., Koch G., Birkhed D., Wendt L.K. (2013). Caries prevalence in Swedish 20-year-olds in relation to their previous caries experience. Caries Res.

[5] Braun P.A., Quissell D.O., Henderson W.G., Bryant L.L., Gregorich S.E., George C. (2016). A cluster-randomized, community-based, tribally delivered oral health promotion trial in Navajo head start children. J Dent Res.

[6] First Nations Information Governance Centre (2012). Report on the findings of the First Nations Oral Health Survey (FNOHS) 2009–10.

[7] Ministry of Health (2010). Our oral health: key findings of the 2009 New Zealand Oral Health Survey.

[8] Do L.G., Spencer A.J. (2016). Oral health of Australian children: the National Child Oral Health Study 2012–14.

[9] American Academy of Pediatric Dentistry (2010). Definition of early childhood caries (ECC). Pediatr Dent.

[10] Li Y., Caufield P.W., Dasanayake (2005). Mode of delivery and other maternal factors influence the acquisition of *Streptococcus mutans* in infants. J Dent Res.

[11] Finlayson T.L., Gupta A., Ramos-Gomez F.J. (2017). Prenatal maternal factors, intergenerational transmission of disease, and child oral health outcomes. Dent Clin N Am.

[12] Chaffee B.W., Gansky S.A., Weintraub J.A. (2014). Maternal oral bacterial levels predict early childhood caries development. J Dent Res.

[13] Weintraub J.A., Ramos-Gomez F., Jue B., Shain S., Hoover C.I., Featherstone J.D. (2006). Fluoride varnish efficacy in preventing early childhood caries. J Dent Res.

[14] Lawrence H.P., Binguis D., Douglas J., McKeown L., Switzer B., Figueiredo R. (2008). A 2-year community-randomized controlled trial of fluoride varnish to prevent early childhood caries in Aboriginal children. Community Dent Oral Epidemiol.

[15] Slade G.D., Bailie R.S., Roberts-Thomson K., Leach A.J., Raye I., Endean C. (2011). Effect of health promotion and fluoride varnish on dental caries among Australian Aboriginal children: results from a community-randomized controlled trial. Community Dent Oral Epidemiol.

[16] Nowak A.J., Casamassimo P.S. (1995). Using anticipatory guidance to provide early dental intervention. J Am Dent Assoc.

[17] Plutzer K., Spencer A.J. (2008). Efficacy of an oral health promotion intervention in the prevention of early childhood caries. Community Dent Oral Epidemiol.

[18] Plutzer K., Spencer A.J., Keirse M.J. (2012). Reassessment at 6–7 years of age of a randomized controlled trial initiated before birth to prevent early childhood caries. Community Dent Oral Epidemiol.

[19] Harrison R., Benton T., Everson-Stewart S. (2007). Effect of motivational interviewing on rates of early childhood caries: a randomized trial. Pediatr Dent.

[20] Finlayson T.L. (2017). Limited evidence shows that a motivational interviewing approach may be the most effective behavioral intervention for reducing dental caries in children. J Evid Based Dent Pract.

[21] Government of South Australia (2013). Pregnancy outcome in South Australia 2011.

[22] Merrick J., Chong A., Parker E., Roberts-Thomson K., Misan G., Spencer J. (2012). Reducing disease burden and health inequalities arising from chronic disease among Indigenous children: an early childhood caries intervention. BMC Public Health.

[23] American Academy of Pediatric Dentistry (2016). Guideline on periodicity of examination, preventive dental services, anticipatory guidance/counseling, and oral treatment for infants, children, and adolescents. Pediatr Dent.

[24] Venner K.L., Feldstein S.W., Tafoya N. (2006). Native American motivational interviewing: weaving native American and western practices.

[25] Jamieson L., Bradshaw J., Lawrence H. (2016). Fidelity of motivational interviewing in an early childhood caries intervention involving indigenous Australian mothers. J Health Care Poor Underserved.

[32] Lalloo R., Jamieson L.M., Ha D. (2016). Inequalities in tooth decay in Australian children by neighbourhood characteristics and indigenous status. J Health Care Poor Underserved.

[33] Williams S.D., Parker E.D., Jamieson L.M. (2010). Oral health-related quality of life among rural-dwelling indigenous Australians. Aust Dent J.

[34] Roberts-Thomson K.F., Spencer A.J., Jamieson L.M. (2008). Oral health of Aboriginal and Torres Strait Islander Australians. Med J Aust.

[35] Jamieson L.M., Parker E.J., Armfield J.M. (2007). Indigenous child oral health at a regional and state level. J Paediatr Child Health.

[36] Marinho V.C., Worthington H.V., Walsh T. (2013). Fluoride varnishes for preventing dental caries in children and adolescents. Cochrane Database Syst Rev.

[37] de Silva-Sanigorski A., Ashbolt R., Green J., Calache H., Keith B., Riggs E. (2013). Parental self-efficacy and oral health-related knowledge are associated with parent and child oral health behaviors and self-reported oral health status. Community Dent Oral Epidemiol.

[38] Berkman N.D., Sheridan S.L., Donahue K.E., Halpern D.J., Viera A., Crotty K. (2011). Health literacy interventions and outcomes: an updated systematic review. Evid Rep Technol Assess.

[39] Kay E.J., Locker D. (1996). Is dental health education effective? A systematic review of current evidence. Community Dent Oral Epidemiol.

[40] Wyne A.H. (1990). Prevalence and risk factors for nursing caries in Adelaide pre-school children. Child, adolescent and family – professional digest.

[41] Warren J.J., Kramer K.W., Phipps K., Starr D., Dawson D.V., Marshall T. (2012). Dental caries in a cohort of very young American Indian children. J Public Health Dent.

[42] Phipps K.R., Ricks T.L., Manz M.C. (2012). Prevalence and severity of dental caries among American Indian and Alaska Native preschool children. J Pub Health Dent.

[43] Borrelli B., Tooley E.M., Scott-Sheldon L.A.J. (2015). Motivational interviewing for parent-child health interventions: a systematic review and meta-analysis. Pediatr Dent.

[44] de Silva A.M., Hegde S., Akudo Nwagbara B., Marshall T., Calache H., Gussy M.G. (2016). Community-based population-level interventions for promoting child oral health. Cochrane Database Syst Rev.

