# Supplementary material for: Dental Disease Outcomes Following a 2-Year Oral Health Promotion Program for Australian Aboriginal Children and Their Families: A 2-Arm Parallel, Single-blind, Randomised Controlled Trial
Source: eClinicalMedicine. 2018 Jul 23;1:43–50. doi: 10.1016/j.eclinm.2018.05.001 (PMC6537568; doi:10.1016/j.eclinm.2018.05.001)
Supplement: Supplementary table — Baseline sample characteristics by follow-up and loss-to-follow-up children at 2-year examinations. [file mmc1.docx]

***Supplementary Table*: Baseline sample characteristics by follow-up and loss-to-follow-up children at 2-year examinations**

|  | **Fellow-up** | | | |  | **Loss to follow-up** | | | |
| --- | --- | --- | --- | --- | --- | --- | --- | --- | --- |
|  | **N (%)** | | | **p-value** |  | **N (%)** | | | **p-value** |
|  | Total  (n=324) | Intervention  (n=159) | Control  (n=165) |  |  | Total  (n=112) | Intervention  (n=59) | Control  (n=53) |  |
| **Maternal age** |  |  |  | 0.142 |  |  |  |  | 0.076 |
| 14-24 | 172 (53.1) | 91 (57.2) | 81 (49.1) |  |  | 66 (53.2) | 39 (60.9) | 27 (45.0) |  |
| 25+ | 152 (46.9) | 68 (42.8) | 84 (50.9) |  |  | 58 (46.8) | 25 (39.1) | 33 (55.0) |  |
| **Education** |  |  |  | 0.725 |  |  |  |  | 0.645 |
| High school or less | 226 (70.0) | 112 (70.9) | 114 (69.1) |  |  | 88 (79.3) | 45 (77.6) | 43 (81.1) |  |
| Trade or University | 97 (30.0) | 46 (29.1) | 51 (30.9) |  |  | 23 (20.7) | 13 (22.4) | 10 (18.9) |  |
| **Income** |  |  |  | 0.858 |  |  |  |  | 0.374 |
| Job | 56 (17.34) | 28 (17.72) | 28 (17.0) |  |  | 4 (3.7) | 3 (5.2) | 1 (2.0) |  |
| Centrelink | 267 (82.7) | 130 (82.3) | 137 (83.0) |  |  | 105 (96.3) | 55 (94.8) | 50 (98.0) |  |
| **HCC status** |  |  |  | 0.738 |  |  |  |  | 0.127 |
| Yes | 254 (79.9) | 125 (80.7) | 129 (79.1) |  |  | 95 (89.6) | 46 (85.2) | 49 (94.2) |  |
| No | 64 (20.1) | 30 (19.4) | 34 (20.9) |  |  | 11 (10.4) | 8 (14.8) | 3 (5.8) |  |
| **Residential location** |  |  |  | 0.320 |  |  |  |  | 0.478 |
| Metropolitan | 116 (36.3) | 53 (33.5) | 63 (38.9) |  |  | 55 (45.1) | 26 (41.9) | 29 (48.3) |  |
| Non- metropolitan | 204 (63.8) | 105 (66.5) | 99 (61.1) |  |  | 67 (54.9) | 36 (58.1) | 31 (51.7) |  |
| **Usual reason visit dentist** |  |  |  | 0.740 |  |  |  |  | 0.922 |
| Problem | 195 (61.9) | 98 (62.8) | 97 (61.0) |  |  | 72 (69.2) | 42 (71.2) | 37 (68.5) |  |
| Check-up | 120 (38.1) | 58 (37.2) | 62 (39.0) |  |  | 32 (30.8) | 17 (28.8) | 17 (31.5) |  |
| **Brush yesterday** |  |  |  | 0.748 |  |  |  |  | 0.968 |
| Yes | 239 (76.0) | 116 (75.8) | 123 (77.4) |  |  | 74 (69.8) | 39 (69.6) | 35 (70.0) |  |
| No | 73 (23.4) | 37 (24.2) | 36 (22.6) |  |  | 32 (30.2) | 17 (30.4) | 15 (30.0) |  |
| **Self-rated oral health** |  |  |  | 0.072 |  |  |  |  | 0.155 |
| Excellent, very good or good | 153 (47.2) | 67 (42.1) | 86 (52.1) |  |  | 43 (38.4) | 19 (32.2) | 24 (45.3) |  |
| Fair or poor | 171 (52.8) | 92 (57.9) | 79 (47.9) |  |  | 69 (61.6) | 40 (67.8) | 29 (54.7) |  |
| **Self-rated general health** |  |  |  | 0.480 |  |  |  |  | 0.542 |
| Excellent, very good or good | 294 (91.0) | 142 (89.9) | 152 (92.1) |  |  | 97 (86.6) | 50 (84.8) | 47 (88.7) |  |
| Fair or poor | 29 (9.0) | 16 (10.1) | 13 (7.9) |  |  | 15 (13.4) | 9 (15.2) | 6 (11.3) |  |
